# Electroacupuncture as an Adjunctive Therapy for Bell’s Palsy: A Case Report

**DOI:** 10.7759/cureus.112675

**Published:** 2026-07-14

**Authors:** Mark Jasper S Torres, Isabella Lai

**Affiliations:** 1 Integrative Medicine, Infinity Wellness Institute, Los Angeles, USA

**Keywords:** acupuncture therapy, asymmetric facial paralysis, bell`s palsy, ea electroacupuncture, electroacupuncture, facial nerve paralysis, idiopathic facial paralysis

## Abstract

Bell’s palsy is the most common cause of acute unilateral lower motor neuron-type facial nerve paralysis and is generally managed with early administration of corticosteroids, with or without antiviral therapy. Although most patients experience substantial recovery, a clinically meaningful subset-including older adults, individuals with delayed presentation, and those with comorbid medical conditions-may be left with incomplete resolution, with persistent facial weakness, asymmetry, or synkinesis. Electroacupuncture (EA) has been proposed as an adjunctive treatment modality for facial nerve recovery, with hypothesized mechanisms including enhanced neuromuscular activation, improved regional blood flow, modulation of local inflammatory responses, and facilitation of peripheral nerve regeneration. However, robust clinical evidence and detailed case-based documentation remain limited.

We report the case of an 81-year-old male patient who presented with acute left-sided lower motor neuron-type facial paralysis. The diagnosis was consistent with Bell’s palsy, after a cerebrovascular accident was excluded, and neuroimaging demonstrated no acute intracranial pathology. An integrative treatment approach incorporating acupuncture with low-frequency electrical stimulation was initiated on day 17 post-onset, targeting facial and cranial acupoints. EA treatment was administered twice weekly for seven weeks and was supplemented with conservative home-based interventions. Facial nerve function improved from House-Brackmann Grade IV at presentation to Grade I-II.

While this case suggests that EA may have a role as an adjunctive therapy in Bell’s palsy, including in patients with delayed presentation and less favorable prognostic features, its interpretation is limited by the single-case design and the known potential for spontaneous recovery. Accordingly, controlled prospective studies are warranted to further evaluate the efficacy, optimal timing, and clinical utility of EA in the management of acute facial nerve paralysis.

## Introduction

Bell’s palsy is the leading cause of facial nerve paralysis, marked by the sudden loss of motor control on one side of the face. Annual incidence estimates range from 15 to 30 cases per 100,000 population, accounting for 60%-75% of all unilateral facial palsy cases [1-3]. Although Bell’s palsy is considered idiopathic, inflammation and swelling of the seventh cranial nerve may contribute significantly to nerve dysfunction [1,2]. The reactivation of latent herpes simplex virus type 1 (HSV-1) and SARS-CoV-2 during the COVID-19 pandemic has also demonstrated strong associations between infection and Bell’s palsy development [3-5].

Current treatment guidelines recommend oral corticosteroids, with optimal treatment efficacy achieved when initiated within 72 hours of symptom onset [1,6]. The addition of antiviral agents to corticosteroid therapy may also provide additional benefit, particularly in reducing synkinesis [1,6-8]. Despite a positive prognosis and appropriate care, approximately 30% of patients still develop persistent sequelae, including permanent facial paresis and facial asymmetry [1,2]. Additional complications of Bell’s palsy may include lagophthalmos, which increases the risk of corneal abrasion and exposure keratopathy; therefore, prophylactic eye care is recommended [1-3].

Persistent functional deficits following Bell’s palsy have prompted interest in complementary therapeutic approaches, such as acupuncture and electroacupuncture (EA). Several mechanisms have been proposed to explain how acupuncture may support facial nerve recovery, including enhanced neural connectivity and increased activity in the primary sensory cortex and primary motor cortices. These neuroplastic changes may strengthen sensorimotor processing and cortical reorganization after peripheral facial nerve injury, thereby supporting recovery of facial motor function [9]. EA may further augment these therapeutic effects through controlled electrical stimulation that enhances neuromuscular activation [10].

Despite this growing body of evidence supporting acupuncture as an adjunctive therapy for Bell's palsy, detailed case reports documenting the clinical course and outcomes of EA treatment remain limited. Here, we present the case of an elderly patient with Bell's palsy who was treated with an integrative EA protocol, performed twice a week for 25 minutes over seven weeks, associated with progressive improvement in facial symmetry and voluntary facial movement.

## Case presentation

An 81-year-old male patient with a history of polymyalgia rheumatica, arthritis, and bilateral vestibular hypofunction presented with deviation of the angle of the mouth to the right side and diminished activation of left facial musculature for 2-3 weeks. The patient reported waking up with left lateral facial paralysis and an aching sensation radiating from behind the left ear to the left jaw and neck. He visited the emergency room for evaluation and was diagnosed with Bell’s palsy. An MRI brain scan showed no acute infarction or intracranial pathology. He was prescribed a course of prednisone 60 mg, valacyclovir, and erythromycin ointment for dry eyes, which he completed with minimal improvement. He retained normal tactile sensation but had incomplete motor control of the left lateral facial muscles and persistent left-sided pain. He denied having any similar events or facial symptoms in the past.

Prior to the onset of Bell’s palsy, the patient experienced recurring neck stiffness, chronic low back pain, constant dizziness, and intermittent lightheadedness related to his underlying conditions. Additionally, he reported moderate fatigue, as the onset of left ear pain had been disrupting his sleep quality, and that his current health condition had caused some stress.

**Figure 1:**
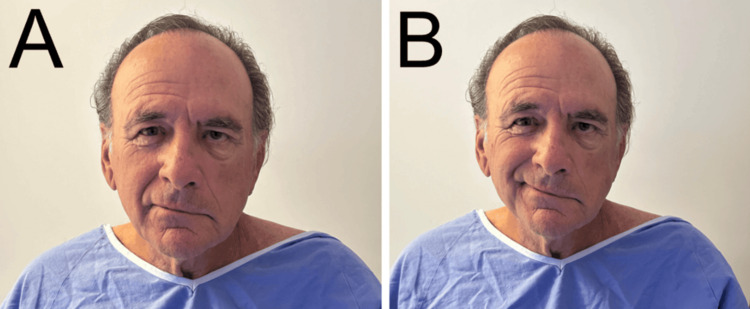
Baseline facial nerve dysfunction (House-Brackmann Grade IV). (A) Resting facial asymmetry demonstrating left-sided facial droop and flattening of the nasolabial fold. (B) Attempted smile demonstrating reduced elevation of the left oral commissure and diminished activation of the left facial musculature. Written informed consent for the publication of these images was obtained from the patient.

At day 17 following symptom onset and referral from the patient’s physical therapist, the patient presented to the outpatient clinic for evaluation. Physical examination demonstrated left-sided facial weakness with facial droop and reduced activation of the orbicularis oris and orbicularis oculi muscles. There was decreased elevation of the left oral commissure during attempted smiling, accompanied by diminished voluntary movement of the affected facial musculature. Mild resting asymmetry was present, with reduced movement of the left side of the mouth compared with the contralateral side. Facial nerve dysfunction was classified as House-Brackmann Grade IV by the treating physician based on clinical examination and photographic assessment, consistent with moderately severe facial weakness. Sensation was intact to light touch. Mild tenderness was noted posterior to the left ear and along the masseter muscle. Vital signs were within normal limits, and the remainder of the neurological examination was unremarkable. Clinical photographs obtained at the initial evaluation demonstrated left-sided facial weakness with resting asymmetry and impaired voluntary facial movement (Figure 1).

**Figure 2:**
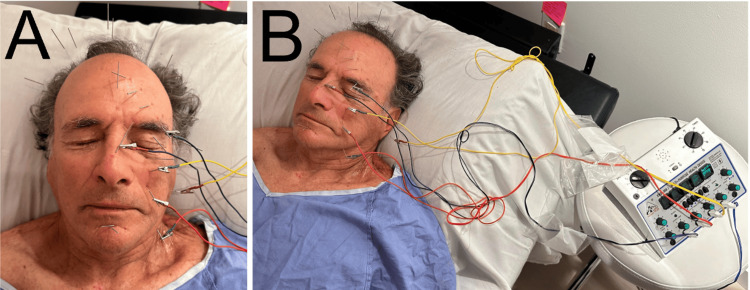
Acupuncture and electroacupuncture protocol. (A) Acupuncture needles were placed along cranial and facial acupoints corresponding to the affected facial nerve distribution. (B) Electrical stimulation was applied to the ipsilateral Stomach meridian points (ST1-ST6) to promote neuromuscular activation and regional circulation. Written informed consent for the publication of these images was obtained from the patient.

Given the patient’s age, medical comorbidities, and persistent facial dysfunction, an integrative treatment approach utilizing acupuncture with electrical stimulation was initiated. Electrical stimulation was performed using a KWD-808I Multi-Purpose Health Device (Changzhou Yingdi Electronic Medical Device Co., Ltd, Jiangsu, China), an EA unit designed to provide low-frequency electrical stimulation through acupuncture needles. Electrical stimulation was delivered at 2 Hz, with intensity adjusted to patient tolerance, for 25 minutes. Acupuncture needles sized 0.18 × 15 mm were placed at the following points: Du20 (Baihui), Yin Tang, Bi Tong, Yuyao, Triple Heater 21 (TH21, Ermen), Small Intestine 19 (SI19, Tinggong), and Gallbladder 2 (GB2, Tinghui). Electrical stimulation was applied to the following acupuncture points using needles sized 0.20 × 30 mm: Stomach 1 (ST1, Chengqi), Stomach 2 (ST2, Sibai), Stomach 3 (ST3, Juliao), Stomach 4 (ST4, Dicang), Stomach 5 (ST5, Daying), and Stomach 6 (ST6, Jiache) (Figure 2).

Facial acupoints along the Stomach meridian were selected because they overlie muscles innervated by branches of the facial nerve and are commonly used in Bell's palsy treatment protocols. Cranial points were selected to support regional circulation and neuromuscular activation.

EA treatment was scheduled for 25 minutes twice weekly for the following seven weeks, depending on patient improvement. For at-home care, the patient was instructed to apply warm compresses to the left jaw and ear nightly and avoid overexertion or cold exposure to the affected side. The patient was also recommended to continue gentle massage and participate in physical therapy.

**Figure 3:**
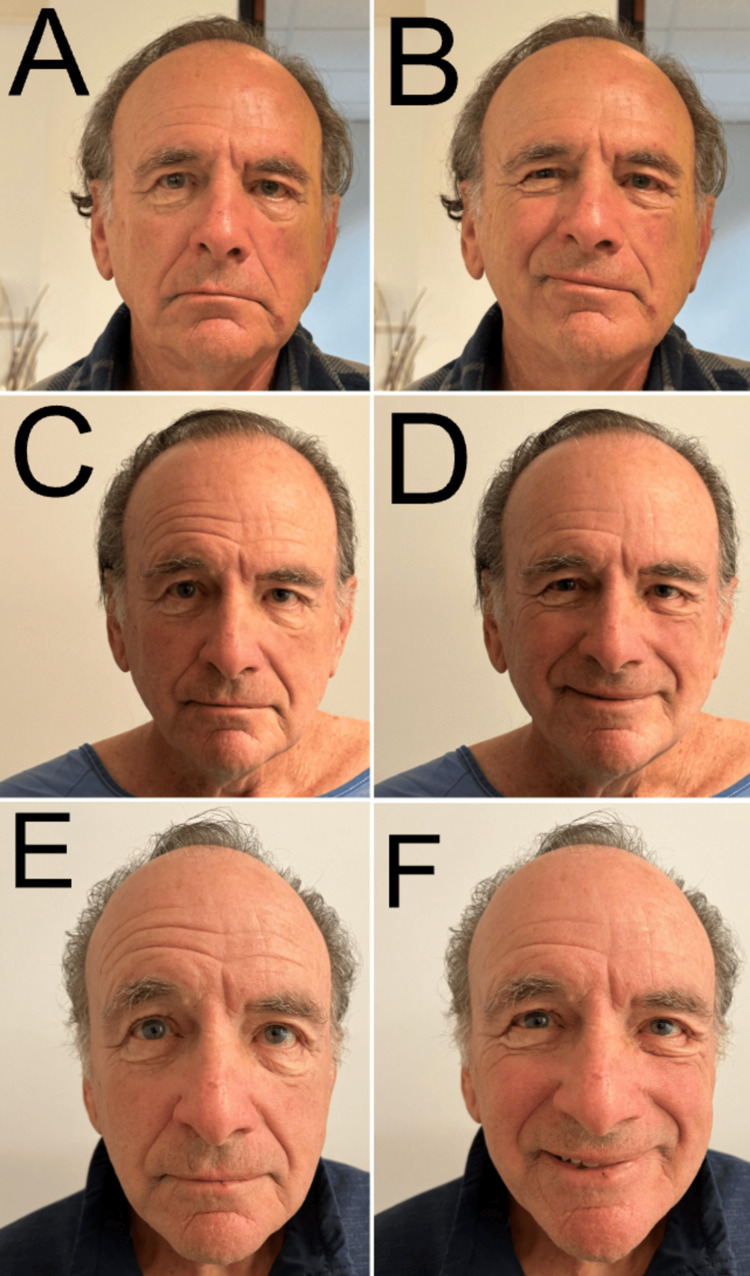
Serial photographic assessment demonstrating progressive improvement in facial nerve function during electroacupuncture treatment. (A, B) Approximately 1 week after treatment initiation (2 sessions), House-Brackmann Grade III facial nerve dysfunction with persistent mild resting asymmetry and improved voluntary movement of the left oral commissure. (C, D) Approximately 5 weeks after treatment initiation, House-Brackmann Grade II facial nerve dysfunction with near-symmetric facial appearance and substantial recovery of voluntary facial movement. (E, F) Approximately 7 weeks after treatment initiation, House-Brackmann Grade I-II facial nerve function with symmetric facial posture at rest and near-complete recovery of smile symmetry. Written informed consent for the publication of these images was obtained from the patient.

House-Brackmann grading was performed by a single treating physician at each follow-up visit using serial clinical examinations and photographic documentation. Facial nerve function improved from House-Brackmann Grade IV at presentation to Grade III after two treatment sessions, Grade II by approximately five weeks, and Grade I-II at completion of the seven-week treatment course (Table 1). Early improvement was noted after one week and two treatment sessions, with increased elevation of the left oral commissure and reduced resting asymmetry compared with baseline (Figures 3A, 3B). By approximately week five, the patient reported near-complete recovery of Bell’s palsy symptoms and regained the ability to smile voluntarily (Figures 3C, 3D). At the conclusion of the seven-week treatment course, the patient demonstrated significant improvement in facial symmetry and voluntary movement compared with baseline, with minimal residual deficits noted only during maximal effort (Figures 3E, 3F). No adverse effects related to acupuncture or EA were reported during the treatment period.

**Table 1:** Clinical timeline of Bell's palsy presentation and treatment course.

Time from symptom onset	Clinical event
Day 0	Onset of left-sided facial paralysis
Day 1	Emergency department evaluation. Stroke ruled out. MRI negative. Prednisone, valacyclovir, and erythromycin ointment initiated
Day 17	Initial electroacupuncture evaluation (House-Brackmann Grade IV)
Week 1 (2 treatments)	House-Brackmann Grade III
Week 5	House-Brackmann Grade II
Week 7	House-Brackmann Grade I-II

This study is a single-patient case report and does not meet the definition of human subjects research requiring Institutional Review Board approval. Written informed consent and HIPAA authorization were obtained from the patient for the publication of clinical information and identifiable facial images. The patient understood that, although efforts were made to protect privacy, anonymity cannot be fully guaranteed.

## Discussion

Bell’s palsy is an acute, idiopathic peripheral facial mononeuropathy. The condition is characterized by the rapid onset of unilateral facial muscle weakness or paralysis [1]. Patients commonly present with facial droop, inability to fully close the affected eye, flattening of the nasolabial fold, and loss of forehead wrinkles [1,2]. Although its precise etiology remains unclear, current evidence suggests that inflammation and edema of the facial nerve within the facial canal contribute to nerve compression and dysfunction, with viral triggers such as herpes simplex virus type 1 (HSV-1) reactivation and, more recently, SARS-CoV-2 infection implicated in disease development [1-5].

Conventional management of Bell’s palsy is centered on reducing facial nerve inflammation and preventing long-term complications. Current clinical guidelines recommend oral corticosteroids as first-line therapy, with treatment initiated within 72 hours of symptom onset to maximize the likelihood of complete functional recovery [1,6]. Prednisone or prednisolone is most commonly prescribed and has been shown to significantly improve facial nerve outcomes compared with no treatment [1,5,6]. Antiviral agents such as acyclovir or valacyclovir may be used in conjunction with corticosteroids, particularly in patients with severe paralysis, given the proposed role of viral reactivation in disease pathogenesis; however, evidence suggests that antivirals alone are ineffective and are strongly discouraged [1,6-8]. Supportive care is also an essential component of treatment, especially in patients with impaired eyelid closure, and includes artificial tears, lubricating ointments, and protective eye measures to reduce the risk of corneal injury and exposure keratopathy [1-3]. Rehabilitation with therapy modalities such as thermal treatment, massage, facial exercise, and combination therapies has been considered, though such treatments alone have not demonstrated significant benefit compared with controls, nor have consistent physical therapy protocols been established [8]. Despite these interventions, a subset of patients continues to experience incomplete recovery and chronic sequelae, prompting interest in adjunctive therapies that may further improve functional outcomes [1,2].

This case describes the use of EA as an adjunctive therapy in an elderly patient with Bell's palsy who achieved near-complete recovery within seven weeks. Although the patient received guideline-directed corticosteroid therapy shortly after symptom onset [11], several clinical factors, including advanced age (81 years), persistent facial deficits at presentation, multiple medical comorbidities, and delayed initiation of EA (day 17), would generally be considered less favorable prognostic features [1,2]. Despite these factors, the patient demonstrated progressive improvement throughout the treatment course, with facial nerve function improving from House-Brackmann Grade IV at presentation to Grade I-II by completion of treatment.

The timing of the acupuncture intervention in this case falls outside the optimal window identified in recent literature. A 2022 propensity score-matched analysis observed that early acupuncture intervention within seven days of symptom onset has the potential to improve recovery time, with a 93.4% favorable outcome rate, compared to 80.3% with delayed treatment [12,13]. Evidence from multiple systematic reviews suggests that acupuncture is associated with improved cure rates and overall treatment effectiveness, compared with pharmacotherapy alone [14,15]. A 2025 meta-analysis that evaluated EA as an adjunctive therapy in patients with refractory facial paralysis suggested that acute-phase acupuncture intervention may help promote axon growth and nerve repair and is associated with an 11% relative increase in the probability of treatment success (RR 1.11) [13,15]. Despite delayed treatment, the patient achieved near-complete recovery by week five, suggesting that EA may remain a reasonable adjunctive consideration beyond the acute phase, particularly when combined with standard pharmacotherapy.

The proposed mechanisms underlying the observed effects of acupuncture in Bell's palsy include increased neural excitability, promotion of nerve fiber regeneration, enhanced muscle contraction, and improved blood and lymphatic circulation [8-10]. A 2020 randomized controlled trial using laser speckle contrast imaging demonstrated that manipulative acupuncture significantly improved facial blood perfusion in patients with severe Bell's palsy, with a recovery rate of 91.7% at six months, compared with 78.0% with simple acupuncture [16]. Electrical stimulation through EA has been proposed to further augment these effects by providing controlled neuromuscular activation. A 2023 meta-analysis of EA for intractable facial paralysis reported significantly improved total effective rates (RR 1.23) and cure rates (RR 2.04) compared with non-EA interventions [10]. A 2020 study comparing EA added to standard therapy versus standard therapy alone found significantly higher rates of normal nerve function on electromyography (66.7% vs. 25.0%) and better House-Brackmann grades at 12 weeks (95.8% vs. 50.0% achieving grade ≤ 2) [17].

This case report describes the clinical course of an elderly patient with Bell’s palsy who presented with multiple factors generally associated with less favorable recovery, including advanced age, medical comorbidities, and delayed initiation of adjunctive therapy [1,2]. The report provides detailed documentation of the EA protocol, treatment timeline, and serial photographic assessments that illustrate progressive changes in facial symmetry and voluntary movement over time. These features contribute practical clinical information regarding the implementation of EA as part of an integrative treatment approach.

However, several limitations of this case report warrant consideration. As a single case report, these findings cannot establish causality or generalizability, nor can they determine the independent effect of EA. Although facial nerve function improved from House-Brackmann Grade IV to Grade I-II during the treatment period, spontaneous recovery remains a potential explanation for the observed outcome. Furthermore, the patient completed guideline-directed pharmacologic therapy prior to the initiation of EA. Additional facial outcome measures such as the Sunnybrook Facial Grading System, Facial Disability Index, and electrophysiologic testing were also not implemented, limiting the ability to objectively quantify recovery and compare outcomes across studies. There was also an absence of long-term follow-up, precluding assessment of the durability of treatment effects and the potential for delayed recurrence. In addition, the existing evidence supporting EA for Bell's palsy remains limited by the overall low methodological quality of available studies, including small sample sizes, heterogeneous treatment protocols, inconsistent outcome measures, and variable risk of bias, making definitive conclusions regarding efficacy difficult. Controlled studies are needed to better define the efficacy and clinical role of EA in Bell’s palsy.

Future research should focus on well-designed, adequately powered randomized controlled trials comparing EA as an adjunct to standard therapy versus standard therapy alone, with standardized outcome measures and long-term follow-up. Particular attention should be given to the optimal timing of intervention, treatment frequency and duration, and the identification of patient subgroups most likely to benefit from this integrative approach. Investigation of the neurophysiologic mechanisms underlying acupuncture's effects, including additional functional neuroimaging studies, may provide further insights into its therapeutic potential [8,9].

## Conclusions

This case describes improvement in facial nerve function from House-Brackmann Grade IV to Grade I-II during a seven-week course of EA in an elderly patient with Bell's palsy. Although spontaneous recovery and prior pharmacologic treatment must be considered, EA may represent a useful adjunctive therapy in selected patients. Further prospective studies are needed to clarify its efficacy, optimal timing, and role in the management of Bell’s palsy.
